# Need Assessment to Incorporate Geriatric Oral Health Care Education in New Zealand Nursing Curricula: A Narrative Review Study

**DOI:** 10.1155/ijod/9351150

**Published:** 2025-07-31

**Authors:** Arthi Veerasamy, Karl Lyons, Ian Crabtree, Jithendra Ratnayake, Paul Brunton

**Affiliations:** ^1^Faculty of Dentistry, University of Otago, P. O. Box- 56, Dunedin 9054, New Zealand; ^2^School of Nursing, Otago Polytechnic, Fourth Street Private Bag 1910, Dunedin 9054, New Zealand; ^3^Office of the Deputy Vice-Chancellor, Curtin University, Kent Street, Bentley, Western Australia 6102, Australia

**Keywords:** geriatric oral health, oral health care in nursing, oral health education, oral health in long term care, oral-systemic connection

## Abstract

**Background:** New Zealand (NZ) has almost 58,206 registered nurses who are involved in the care of patients. One group of which are elderly patients being cared for at home or in aged care facilities. Maintenance of oral health and prevention of deterioration in oral health can prevent life-threatening infections and provide greater function and comfort for the older population. Therefore, the aim of this review was to investigate the need for oral health education both theory and practice in nursing curricula in NZ.

**Methods:** A narrative review was conducted following the scale for the quality assessment of narrative review articles (SANRA). A broad literature search was conducted in Medline, Pubmed, Web of Science and Ovid, Scopus, Index New Zealand, Science Direct, Proquest, Proquest Dissertation and Thesis, the Cochrane Library and major health organisation websites. The themes were generated to identify issues requiring a need for oral health education in NZ.

**Results:** The literature review identified 11 problems which necessitates the need for the oral health care education in nursing curriculum.

**Conclusion:** It is concluded that the increased economic burden in older adult care, the increased need for oral health care for elderly populations and increasing treatment needs among the older population demand more sophisticated oral care for the hospitalised elderly population and those in residential care. The inclusion of an elderly oral health care education module into the undergraduate nursing curriculum could improve the oral health outcome of the elderly population.

## 1. Introduction

Recent studies have identified the relationship between oral health and an individual's general health [1–3]. Maintenance of oral health and prevention of deterioration in oral health can prevent life-threatening infections and provide greater function and comfort. However, the importance of oral health has not been embraced by the majority of the population. This is in part, due to a lack of promotion of the advantages of good oral health by non-dental health professional groups, [4, 5] one of which is, registered nurses in practice [6–8].

New Zealand (NZ) has almost 58,206 registered nurses who are involved in the care of patients. One group is elderly patients cared for at home or in aged care facilities [9]. In oral health care, maintenance and disease prevention are not prioritised in nursing education or practice [10]. As a result, nursing students may receive insufficient oral health education, which could negatively impact overall patient care [11]. The period and cohort analysis for the NZ population suggested that 25 years of one's life is lived in poor health and multimorbidity affects one in four adults in NZ [12]. On average, older women in NZ required assistance to live for about 16.7 years and 14.3 years for males. The number of years of care is higher for Māori, Pacific people and those with intellectual disability [13].

Therefore, developing a core set of oral health competencies and associated curricula for undergraduate nurses would enhance their role in oral health promotion and disease prevention. This would then be beneficial to patients and overall lead to an improvement in general health outcomes.

Therefore, this review aims to investigate the need for oral health education, both theory and practice, in nursing curricula in NZ.

## 2. Methods

A narrative review is a comprehensive, critical and objective analysis of the current knowledge on a topic to understand the problem areas, theoretical framework and context of research in the specific topic area. This narrative review was structured and based on widely accepted medical writing guidelines provided by Ferrari [14], the scale for the quality assessment of narrative review articles (SANRA) to increase the feasibility of this review report [15].

The literature review was conducted using the following bibliographic databases: Medline, Pubmed, Web of Science and Ovid, Scopus, Index New Zealand, Science Direct, Proquest, Proquest Dissertation and Thesis and the Cochrane Library. The following internet searches were undertaken: Google, Google Scholar, World Health Organisation Publications, NZ Ministries of Health and Education websites and the Dental Council (NZ) website.

As this is a narrative review the literature search was done in two stages.

First, general search terms such as oral health of elderly, oral health care at residential homes, oral health care knowledge of nurses/caregivers, geriatric oral health education/training and oral health of the elderly population were built into the search strategy. Later themes were identified regarding the research question, for example, the need for oral health care education in undergraduate nursing curricula.

A second detailed literature search was then conducted on identified themes namely, the growing ageing population, the impact of ageing on health and the oral health workforce, the decline in informal care for elderly population, baby boomers' knowledge and expectations, the lack of oral health care protocols in residential homes, dental challenges for the ageing population, oral-systemic health connection, restricted access to oral health services for elderly patients, barriers to oral care in geriatric care and the lack of oral health knowledge among care facility staff.

The review had the following inclusion and exclusion criteria:

Inclusion:

• Manuscripts published in a peer reviewed journal.
• Manuscripts which discussed the oral health of geriatric populations, in residential facilities and in countries in the organisation for economic co-operation and development (OECD).

Exclusion:

• Manuscripts from countries which did not have organised residential facilities for their elderly population.
• Manuscripts that referred to dental nurses rather than general nurses.
• Manuscripts that considered oral health education and prevention for other priority groups.

## 3. Results

In the first literature review stage, 11 problem areas which described the need for oral health care education in nursing curriculum were identified.

1. Growing ageing population.
2. Implication of ageing in the health and oral health workforce.
3. Decline in informal care for elderly population.
4. Baby boomers' knowledge and expectations.
5. Lack of oral health care protocol in NZ residential facilities.
6. Dental challenges for aged populations.
7. Oral-systemic health connection.
8. Lack of access to oral health services.
9. Barriers to oral care in geriatric care.
10. Lack of oral health knowledge among care facility staff.
11. Increase in internationally qualified nurses in the workforce.

A further literature review was conducted to discuss the identified themes in the NZ health and oral health care context.

## 4. Discussion

Identified literature were synthesised into 11 different themes and published peer reviewed literature in NZ and the international literature found in comparative countries with similar health workforce and populations are discussed as problem statements.

### 4.1. Problem Statement 1: The Growing Ageing Population

The world population aged 60 years or over has grown from 382 million in 1980 to 962 million in 2017. The figure is expected to double again by reaching 2.1 billion in 2050 (United Nations, 2017) [16]. The older population is substantially increased in Oceania and many other westernised countries [17]. At the beginning of the 20^th^ century, children out numbered the aged population by eight to one in NZ. Combined reduction of childhood mortality and increased life expectancy for almost 20 years were essential elements for the growth of the aged population in NZ [18] and as a result, NZ is expected to experience a significant increase in the aged population. The percentage of people aged 65 and over is expected to increase from 15% in 2016 to 26% in 2043 ([19]; Statistics New [20]). The recent report from Ministry of Housing and Urban Development [21] suggests that the senior population will grow from 17% (850,000) to 24% (1.5 million) in the next 30 years [22]. Within this age group, the proportion of those aged 80 years and above are expected to increase from the current 25% to 40% in 2050 (Statistics New [20]) and it is predicted that about half of the elderly population will enter a rest home or long-term care at some stage of their life [11, 19, 23]. The report from New Zealand Institute of Economic Research Inc (NZIER; 2024) suggested that the 80+ age group will be the largest band among seniors aged 65+ and they will continue to be the largest group of seniors until 2071 [24].

### 4.2. Problem Statement 2: Implication of Ageing in the Health and Oral Health Workforce

The growing ageing population is the greatest challenge facing the healthcare system in NZ and this is the same across the world [16]. Many countries have identified the challenges posed by the population ageing. However, geriatric medicine and long-term care of older people is not prominent in medical training as it is the least popular speciality for health professionals [5]. For countries like NZ, recruiting health care professionals to care for older adults has already become an issue and NZ immigration has listed aged care registered nurses as a long-term skill shortage (NZ [25]). According to 2011 data, aged residential care costs about $800 million per year for taxpayers [23]. The gross domestic product (GDP) spending for aged care is projected to increase from 14.4% today to 20.9% in 2050 and it is predicted that this will increase the burden on the economy [26]. The raw cost of constructing an aged care facility, excluding chattels is between $101,250 and $117,000 per bed and it costs around $126 per person per day in care costs [19]. The Health NZ report in 2024 highlighted that the aged care system is under significant pressure due to a lack of funding, equity concerns, significant variations between regions and inconsistencies in the delivery of care, causing poor health outcomes for the older population in NZ [27].

Thomson [28] suggested that the absolute number of those who are edentulous in NZ would fall from 314,365 in 2011 to 76,300 in 2031, increasing the need for oral health services and oral care for 65–74-year-olds. Thus, the number of older people entering a residential home with dentures would be less common, whilst the number with more complex fixed prostheses will increase in the coming years [28].

The oral health needs for the ageing population differ based on their dependency levels, namely, no dependency, pre-dependency, low dependency, medium dependency and high dependency (World Dental [29]). Wolff [30] suggested that a varied skill mix and wide range of workforce is required to provide effective oral health care for an elderly population with different levels of dependency. In NZ, the level of dependency and life expectancy is strongly associated with gender and ethnicity [3]. Forward planning is needed to meet the shortfalls in the oral health service workforce since it takes some considerable time for the planning and training of health professionals [30].

### 4.3. Problem Statement 3: Decline in Informal Care for Elderly Population

In the USA and the United Kingdom, the major long-term care provider for the elderly is the family. In the United Kingdom, about one in eight adults provide care for an elderly relative [31]. A report by the United Nations [16] on the ageing population suggested that compared to any other developed and developing countries, there is a very high decline in informal family care for older people in Australia and NZ. Almost 75% of the older population of age 65 and above are living independently and a very minimal proportion of them were living with their family and the majority of older people aged 85 and above were living in residential homes [16]. The decline in fertility rate and the reduced number of children/siblings makes this even worse and most of the frail elderly population is expected to require some residential home [16]. Moreover, women used to contribute more to older adults' informal care and the increased representation of women in the workforce has greatly impacted family-based care for the elderly based on the current ageing trends, almost everyone in NZ will depend on residential care at some point in their life. A study by Swain [32] in NZ suggested that the people who are involved in informal care and caring for the elderly in a family setting are experiencing elevated levels of depression and anxiety due to the added financial burden. Moreover, informal carers do not have the expertise or resources to care for frail older people and domestic homes do not have the equipment and furniture to safely care for older people [27]. Hence, for NZ, the residential care system and community living are ideal for the elderly, which makes the nurses/aid workers at a residential home often the main carers for the older population.

### 4.4. Problem Statement 4: Baby Boomers' Knowledge and Expectations

The results of a longitudinal study suggested that successful ageing has changed the expectations of consumers who wish to be more involved in the decision-making process of their healthcare [33]. Compared to older adults of yesteryear, the baby boomers approach their healthcare providers with expectations of wellness and a healthy lifestyle [33]. The baby boomer generation is more highly educated, and they have access to a wider variety of information due to technology and communication development and consequently have a greater awareness of available treatments [18, 33]. The ageing population living independently or going to a residential home would, it is suggested, have higher expectations of their oral health care. In NZ in the 1960s, the fertility rate exceeded four births per woman, a figure considered high on an international scale [34]. Similar to other OCED countries, the ageing baby boomer population will make up an increasingly significant portion of our society [35]. According to Stats NZ, this group is expected to grow to approximately 1 million people, representing 19% of the total population by 2028 [36].

### 4.5. Problem Statement 5: Lack of Oral Health Care Protocol in NZ Residential Homes

In 2016, Kelsen and Thomson [37] examined the written oral healthcare policies in randomly selected care facilities in NZ. One hundred and thirty-nine care facility managers and nurses participated in the study and it was found that only 36% of care facilities had a written, oral care plan for the residents, and only 11% had a baseline oral examination at admission. A written oral health care plan or policies were available in one-third of facilities and among those who reported having a protocol, only half of the facilities had sought help from a dental professional to draft the protocol. Facilities reported that about one-eighth of residents had received oral health care in the past 12 months and on most occasions, the common reason for dental care was a dental emergency. In essence an ambulance at the bottom of the cliff approach. Hence, it would appear that regular dental care is not identified as an important need in most care facilities and nurses are not regularly addressing the oral health needs of the residents. The facilities indicated that a dental emergency or problem would be identified within 48 h of its development, and it took at least 3 days to get an appointment for treatment, which is unacceptable. Almost all the managers who participated in the study indicated that the oral care of residents should be improved. More than half of residents needed assistance to perform their regular oral hygiene care and dementia patients required further help. Schluter et al. [38] suggested that older adults experience financial constraints and access issues when admitted to long-term care, which affects routine dental visits for older adults [38].

The oral health of residents could be improved by involving registered nurses in oral health care by properly training them to provide prevention services, identifying the oral health care needs of the residents by regular oral examination and supporting qualified and unqualified caregivers in providing good oral hygiene care. Almost 90% of nurses and managers responded that oral care of their residents can be improved by training their staff [37]. However, they favoured any initiatives which did not require the facility to pay to train the staff [37]. Hence, a more practical option for NZ is training nursing students to provide oral health care for older people.

### 4.6. Problem Statement 6: Dental Challenges for the Aged Population

Poor oral health amongst older people is largely due to high levels of tooth loss, an increased rate of dental root caries, the high prevalence rate of periodontal (gum) disease, xerostomia (self-reported dry mouth) and oral precancer/cancer conditions [39]. Edentulism is more common among elderly people all over the world. The data from 2005 suggested that there was 26% and 58% of edentulism among those 65 years and older in the USA and Canada, respectively. In NZ, edentulism among 65–74-year-olds was reported to be 72.3% and 58.6% in 1976 and 1988, respectively, and this has reduced to less than 40% in 2009 [28]. Edentulism is strongly associated with sociodemographic variables with rates being higher among females and ethnic minority groups [28]. Carter et al. [40] suggested that compared to one decade ago, more dependent elderly people are retaining their natural teeth, but the health of the teeth has not improved.

A recent national oral health survey [3] was conducted in 120 (*N* = 987) randomly selected aged residential centres in NZ to investigate residents' clinical oral health status and its association with cognitive function and dependency level. The results suggested that those with impaired cognitive function and highly dependent older adults had greater numbers of teeth with decay [3]. The treatment needs for moderately impaired and severely impaired cognitive functions were higher compared to the unimpaired population. The oral debris level was higher amongst those with a higher dependency level due to a lack of self-care or ineffective oral hygiene care from carers. Petersen [41] suggested that institutionalised and homebound elderly had poorer oral health when compared to independent and active elderly due to a lack of oral health care provided on a day-to-day basis. Major oral diseases such as dental caries and periodontitis are highly biofilm (plaque) dependent and perfect debris removal using a toothbrush and floss is recommended to prevent disease progression [37]. Schluter et al. [38] conducted an assessment of 144,380 inter-RAI-home care (HC) assessments completed by 97,229 older adults and 195,549 inter-RAI-long-term care facilities (LTCFs) assessments from 62,798 people. The average age was 82–84 years at their first assessment. Only about 25% of community-dwelling older adults and 17.5% of those in aged care had a dental check in the last year, with Māori having significantly lower rates of recent dental exams than NZ Europeans [38]. Lack of oral health protocol in residential care and improper debris removal leads to increased dental caries among high dependent older adults in NZ [37].

Hyland et al. [42] analysed the same national data and conducted a study investigating the extent of residual dentition among older New Zealanders living in residential aged care. Almost 45% (*n* = 443) of participants (65+) were dentate; about half of them were aged 85+ and, two-thirds were females. The studies by Thomson et al. [3] and Hyland et al. [42] suggested that even though edentulism is declining due to increased knowledge and changing attitudes toward oral health, caries associated with incremental tooth loss is still prevalent among the elderly due to various barriers in maintaining good oral health. Smith [43] suggested that addressing the determinants of oral health and achieving a better oral health outcome could be achieved by utilising the full array of policy action available to the government. An important focus for policy development suggested training of the care facility staff. The NZ Dental Association has been conducting workshops for rest home carers on oral health care, which the Ministry of Health funds. Only 10% of carers received this service due to a lack of funding [44]. In addition to providing training to the current staff at residential centres, providing oral health education to the current nursing students would be ideal and the most economical option.

### 4.7. Problem Statement 7: Oral-Systemic Health Connection

The connection between oral and systemic health could be different for the elderly who retained their teeth (more than 21), partially retained their (fewer than 21) and those who did not (complete edentulism) [45].

There is strong evidence in the literature on the association between oral health quality of life and general health quality of life. Two major studies on 65-year-olds in the United Kingdom and the USA suggested that edentulism is a significant risk for inadequate dietary intake when compared with other dental problems [46, 47]. Individuals with fewer than 21 teeth with or without a prosthesis had limited consumption of fruits, vegetables and protein-rich foods. The intake of carbohydrate-rich food was the most favoured diet for those with fewer than 21 teeth [47]. Malnutrition in the elderly has also been associated with swallowing problems, mainly due to poor mastication, a lack of saliva and, occasionally, organic causes [48]. Although, Bradbury et al. [49] suggested that edentulism affected the quality of life more than it affected the nutritional intake. As previously discussed, the current elderly population is retaining their teeth, but the majority have restorations and at least one prosthesis [50]. Lack of good oral hygiene is common due to reduced manual dexterity in ageing and so the frail or elderly population depends highly on caregivers for regular oral hygiene procedures [10].

Xerostomia, which is a subjective sensation of oral dryness caused due to salivary gland hypofunction and changes in salivary composition, is another major issue in older adults [51]. A lack of oral hygiene coupled with a dry mouth can cause dental caries and periodontitis. Recent studies have also identified an association between periodontal diseases and diabetes [30], atherosclerosis, Alzheimer's disease [52], cardiovascular disease and myocardial infarction [2]. Meta-analysis and systemic review studies have widely confirmed these associations [1]. Many studies have shown that controlling periodontal disease with oral hygiene procedures and its impact on reducing blood sugar levels [45]. The epidemiological evidence consistently reported that periodontitis increased the incidence of cardiovascular disease [45]. Hence, in a NZ context, potentially suitably trained nurses and caregivers can play important roles in both general and oral hygiene practices and it would be ideal for integrating oral health into general health programmes [41].

### 4.8. Problem Statement 8: Access to Oral Health Services

In NZ, oral health care is funded for children and adolescents under 18 years, and for low-income adults, up to $300 per year is available [53]. There is some funding available for special needs and medically compromised patients, but the Ministry of Health website suggested the amount of funding available to medically compromised patients depends on where they live as funding is through district health boards in NZ which vary [53]. The contract between the District Health Boards of NZ and aged care facilities states that residential homes are responsible for providing access to oral health services and residents are accountable for the costs involved in their oral health care. NZ currently lacks a formal oral health public policy for the aged population, irrespective of their dependency level. Older people highly dependent on carers were 1.6 times more likely not to visit a dental professional than those who live independently [54]. For rural residential care, access to a dentist for regular and emergency care is poor compared to city facilities [37]. As previously discussed, aged care facility managers and nurses believe that lack of funding is a major problem for residents accessing oral health care [10].

### 4.9. Problem Statement 9: Barriers to Oral Care in Geriatric Care

The international literature has identified important barriers for elderly oral health such as:

• Low priority placed by staff on oral care.
• Little or no rebuke, if oral care is omitted.
• Lack of training in oral care provision.
• Negative attitudes towards oral care.
• Few regulations and poor enforcement regarding oral care.

Recent research in NZ has suggested that transport of the resident to a dentist, the willingness of a dentist to treat residents at the nursing care facility, time constraints on facility nursing staff and a lack of interest in dental care by residents, residents' family, nursing staff and general practitioner as barrier to care for older adults' oral health [37]. A qualitative study by Smith et al. [55] suggested barriers at the individual level reducing access to dental services such as the cost of dental treatment, negotiating transport issues, social isolation and health conditions impacting on mobility and access to oral health care. While there is a greater need for improving oral health access to older people in long term care, preventing and managing oral health issues will reduce the burden of diseases. About 905 nurses/managers indicated that residents' oral health could be improved by providing free training to nurses on oral health care to carry out an oral examination and provide oral hygiene care [37]. The economic burden of training nurses after recruiting would put additional pressure on aged care.

### 4.10. Problem Statement 10: Lack of Oral Health Knowledge Among Care Facility Staff

A study was conducted in NZ to understand the knowledge of caregivers, registered nurses, clinical managers and facility managers involved in the long term care for elderly in Nelson and Hawkes Bay regions [11]. Clinical managers, often nurses, and nursing staff all held a Bachelor of Nursing degree or an equivalent qualification. A semi-structured qualitative interview was conducted to understand their personal dental experiences; oral health training obtained; current oral health knowledge; day-to-day facility experiences; the participants' further thoughts on oral health care [11].

The participants had a very minimal knowledge of dental caries and had never received any information regarding periodontal disease, the benefits of fluoride and oral-systemic health relationships. All participants indicated that there is some connection between oral and general health, but none of them were able to describe the relationship [11]. The participants indicated that they are working in a high stress work environment with high expectations that are difficult to meet. Almost all participants suggested that there is a high unmet oral health need for the residents requiring a regular visit by a dental professional. The authors of the study concluded by echoing the recommendations provided by McKelvey et al. [56] and Smith [43] to include oral health care in the curriculum for caregiving and nursing training programmes.

### 4.11. Problem Statement 11: Increase in Internationally Qualified Nurses in the Workforce

The provision of oral hygiene care for the elderly has been increasingly recognised as a priority by international health care bodies, the NZ Dental Association, Aged Care NZ, epidemiologists and researchers.

According to 2019 data, about 10% of nurses were working in a rest home, residential care and elderly continuing care. The number of nurse practitioners has increased from 161 in 2016–370 in 2019 with NZ having the highest number of internationally qualified nurses in OECD countries, making up 26% of the total workforce. Traditionally, most of the overseas qualified nurses came from the United Kingdom, but currently 63% of internationally qualified nurses are from non-OECD countries especially, Philippines (toped in the recent data), India and Sri Lanka [9]. A robust oral health training should be mandatory for nurses from developing countries due to a lack of organised oral health services in their home countries. Currently, some oral health training to care for the elderly is provided by the NZ Dental Association, but it would be advantageous to also offer this training to internationally qualified nurses as part of their orientation to NZ conditions of practice. It is suggested that nursing institutes should take on the responsibility of training their undergraduates to care for oral health among the elderly populations.

## 5. Conclusion

It is concluded that the increased economic burden in older adult care, the increased need for oral health care for elderly populations and increasing treatment needs among the older population demand more sophisticated oral care for the hospitalised elderly population and those in residential care. The inclusion of a geriatric oral health care education module into the undergraduate nursing curriculum could improve the oral health outcome of the elderly population.

## Data Availability

The authors have nothing to report.
